# Phytochemistry and In Vitro Bioactivities of *Elaeagnus angustifolia* L. Leaves: Influence of Solvent Polarity, Antioxidant Properties, and α-Amylase Inhibition

**DOI:** 10.3390/molecules31050861

**Published:** 2026-03-05

**Authors:** Rayene Bouaita, Randa Djemil, Samira Bouhalit, Sabrina Lekmine, Ouided Benslama, Saber Boutellaa, Nabil Touzout, Gema Nieto, Ivan Mustać, Gabrijel Ondrašek, Muhammad Imtiaz Rashid

**Affiliations:** 1 Department of Molecular and Cellular Biology, Faculty of Nature and Life Sciences, University of Abbes Laghrour, Khenchela 40000, Algeria; 2 Laboratory of Biotechnology, Water, Environment and Health (LBWEH), University of Abbes Laghrour, Khenchela 40000, Algeria; 3 Department of Natural and Life Sciences, Faculty of Exact Sciences and Natural and Life Sciences, Larbi Ben M’Hidi University, Oum El Bouaghi 04000, Algeria; 4 Laboratory of Natural Sciences and Materials, Abdelhafid Boussouf University Center, Mila 43000, Algeria; 5 Laboratory Materials and Environment, University Yahia Fares of Medea, Urban Center, Medea 26000, Algeria; 6 Department of Agronomy, Faculty of Sciences, Pole Urban Ouzera, University of Medea, Medea 26000, Algeria; 7 Department of Food Technology, Nutrition and Food Science, Veterinary Faculty, University of Murcia, Regional Campus of International Excellence “Campus Mare Nostrum”, Espinardo, 30100 Murcia, Spain; 8 Department of Soil Amelioration, Faculty of Agriculture, University of Zagreb, Svetošimunska Cesta 25, 10000 Zagreb, Croatia; 9 Center of Excellence in Environmental Studies, King Abdulaziz University, Jeddah 21589, Saudi Arabia

**Keywords:** *Elaeagnus angustifolia* L., phenolic compounds, antioxidant activity, α-amylase inhibition, molecular docking

## Abstract

This study investigated the effect of solvent polarity on extraction yield, phytochemical composition, antioxidant activity, and α-amylase inhibition of *Elaeagnus angustifolia* L. leaf extracts to evaluate their antidiabetic potential. Extraction yields varied with solvent polarity, with the hydroethanolic extract showing the highest (18.00%) and n-hexane the lowest (0.05%) yield. The n-butanol and ethyl acetate fractions contained the most phenolics (309.05 and 290.97 mg GAE/g), ethyl acetate was the richest in flavonoids (102.11 mg QE/g), and tannins were concentrated in dichloromethane (66.24 mg CE/g). HPLC revealed solvent-specific profiles: rutin and gallic acid dominated in n-butanol, quercetin in ethyl acetate, and 4-hydroxybenzoic and ferulic acids in dichloromethane, while chicoric acid appeared in hydroethanolic and n-hexane extracts. Antioxidant assays (DPPH, ABTS, and FRAP) showed strong activity in polar extracts, particularly hydroethanolic and ethyl acetate fractions. The n-hexane extract exhibited the highest α-amylase inhibition (IC_50_ = 36.70 µg/mL), surpassing acarbose (IC_50_ = 126.14 µg/mL), while other fractions were inactive (IC_50_ > 400 µg/mL). Molecular docking highlighted rutin, chlorogenic acid, and chicoric acid as potential enzyme binders. These findings demonstrate the chemical diversity and significant bioactivities of *E. angustifolia* leaves, supporting their potential as natural antidiabetic agents.

## 1. Introduction

An imbalance between reactive oxygen species (ROS) production and the body’s antioxidant defenses results in oxidative stress, which causes cellular damage and plays a critical role in the pathogenesis of various chronic diseases, including diabetes mellitus [1]. The excess ROS can impair insulin signaling pathways and damage pancreatic β-cells, ultimately exacerbating hyperglycemia and diabetic complications [2]. Consequently, mitigating oxidative stress has become a key therapeutic target for management of diabetes [3,4]. In this context, antidiabetic agents that not only regulate blood glucose levels but also possess antioxidant properties are especially valuable, as they can address both glycemic control and oxidative damage, potentially reducing the risk of long-term complications [5,6,7].

Plants have historically been a rich source of bioactive compounds with medicinal properties, including antidiabetic effects [8]. Numerous phytochemicals such as polyphenols, flavonoids, and tannins modulate carbohydrate metabolism enzymes, enhance insulin secretion, improve insulin sensitivity, and scavenge free radicals [9]. The multifaceted biological activities of these natural compounds position them as promising candidates for alternative or complementary therapies in diabetes management. Moreover, the relative safety, accessibility, and longstanding traditional use of medicinal plants support their continued exploration for the development of novel antidiabetic agents.

The effective extraction of these bioactive compounds from plant matrices largely depends on the choice of solvents, as they differ in polarity and thus in their ability to solubilize specific classes of phytochemicals [10,11]. Selecting the appropriate solvent is vital to maximize the recovery of target molecules with desired biological activities while enhancing selectivity and purity [12]. Sequential solvent fractionation with solvents of increasing polarity allows for the separation of complex plant extracts into fractions enriched with distinct phytochemical groups [13]. This strategy aids in bioactivity-guided isolation and facilitates the identification of compounds responsible for the therapeutic effects.

On the other hand, high-performance liquid chromatography (HPLC) is an indispensable analytical technique employed to profile, identify, and quantify phenolic and other bioactive constituents within plant extracts [14,15]. HPLC provides high resolution, sensitivity, and reproducibility, enabling the detailed characterization of phytochemical composition [14,16,17]. This information is crucial for establishing correlations between specific compounds and observed biological activities, quality control, and standardization of herbal preparations, thereby enhancing their acceptance in pharmaceutical applications.

Focusing on *Elaeagnus angustifolia* L., a species belonging to the genus *Elaeagnus*, this plant has been traditionally used in various regions for its medicinal properties, particularly for its anti-inflammatory and antioxidant effects [18,19]. It is known to be rich in secondary metabolites, including flavonoids, phenolic acids, and sesquiterpenes, which contribute to its therapeutic potential [20,21].

In the present study, *E. angustifolia* leaves from the Baghaï region (Khenchela, Algeria) were collected and authenticated to investigate the influence of solvent polarity on extraction yield, phytochemical composition, and associated biological activities. A hydroalcoholic maceration was first performed, followed by sequential liquid–liquid partitioning using solvents of increasing polarity. The obtained fractions were subjected to high-performance liquid chromatography (HPLC) analysis to identify and quantify major bioactive phenolic compounds.

The biological potential of the extracts was evaluated through complementary in vitro assays. Antioxidant activity was assessed using DPPH, ABTS, and FRAP methods, enabling a comprehensive evaluation of radical scavenging and reducing capacities. In parallel, antidiabetic activity was investigated via in vitro α-amylase inhibition and further supported by molecular docking analysis to elucidate the binding interactions between selected phytochemicals and the enzyme active site.

Although *E. angustifolia* has been studied in different geographical regions, no comprehensive phytochemical and biological evaluations have previously been reported for the Algerian population of this species. Considering that environmental, climatic, and edaphic factors significantly influence secondary metabolite biosynthesis, regional ecotypes may exhibit distinct phytochemical fingerprints and bioactivity profiles.

To the best of our knowledge, this study represents the first detailed HPLC-based characterization of phenolic compounds from Algerian *E. angustifolia* leaves, and the first to correlate solvent-dependent extraction patterns with both antioxidant and α-amylase inhibitory activities, supported by molecular docking analysis. This integrative strategy provides novel insights into the pharmacological potential of the Algerian ecotype and expands the existing knowledge on the species within the *Elaeagnaceae* family.

## 2. Results

### 2.1. The Extraction Yields

The extraction yields of *E. angustifolia* leaf powder varied significantly depending on the solvent used for maceration (Table 1). The hydroethanolic solvent produced the highest extraction yield (18%), demonstrating its superior efficiency in extracting bioactive compounds. This was followed by n-butanol, which yielded 4.32%, and ethyl acetate with a yield of 3.84%. The lower-polarity solvents, dichloromethane and n-hexane, resulted in markedly lower extraction yields of 0.32% and 0.05%, respectively. These results underscore the influence of solvent polarity on extraction efficiency, with higher-polarity solvents leading to higher extraction yields.

### 2.2. Total Phenolic Content, Flavonoid Content, and Tannin Content

The quantification of total phenols, flavonoids, and tannins in *E. angustifolia* leaf extracts revealed significant variation depending on the extraction solvent used (Table 2). The n-butanol extract exhibited the highest total phenol content (309.05 ± 0.64 mg GAE/g), followed by the ethyl acetate extract (290.97 ± 1.21 mg GAE/g) and the hydroethanolic extract (244.09 ± 2.07 mg GAE/g), while the n-hexane extract had the lowest phenolic content (92.23 ± 0.65 mg GAE/g). Regarding flavonoids, the ethyl acetate extract showed the highest concentration (102.11 ± 0.79 mg QE/g), whereas the n-butanol extract contained the lowest (29.33 ± 0.58 mg QE/g). For tannins, the dichloromethane (DCM) extract demonstrated the highest content (66.24 ± 0.79 mg CE/g), with hydroethanolic and n-butanol extracts showing moderate levels (25.58 ± 0.36–0.67 mg CE/g). Statistical analysis using one-way ANOVA followed by Dunnett’s post hoc test indicated significant differences among the extracts for all measured parameters (*p* < 0.05), confirming that solvent polarity greatly influences the extraction efficiency of phenolics, flavonoids, and tannins. Overall, polar solvents such as n-butanol, ethyl acetate, and hydroethanolic mixtures were more effective in extracting total phenols and flavonoids compared to non-polar solvents like n-hexane, while DCM selectively extracted high levels of tannins. These results highlight that solvent choice critically affects the yield and composition of bioactive compounds in *E. angustifolia* leaves, which may in turn influence their biological activities.

### 2.3. HPLC Analysis

**Figure 1:**
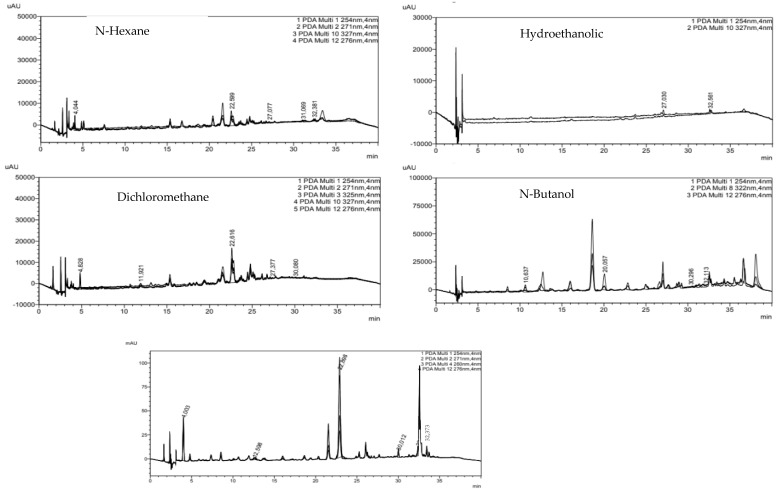
HPLC chromatographic profiles of *E. angustifolia* leaf extracts obtained with different solvents.

Figure 1 and Table 3 depict the phenolic profiles of *E. angustifolia* leaf extracts obtained using five solvents of varying polarity: hydroethanolic, n-hexane, dichloromethane, n-butanol, and ethyl acetate, as determined by high-performance liquid chromatography (HPLC). A comparative analysis of the extracts revealed clear differences in their phenolic compositions, which corresponded to solvent polarity and chemical affinity.

The n-butanol extract delivered the highest concentration of rutin (75.77 mg/g), substantially exceeding that of ethyl acetate extract (15.25 mg/g), whereas rutin was not detected in the hydroethanolic, n-hexane, or dichloromethane extracts. This suggests that n-butanol effectively solubilizes the relatively polar molecular structure of rutin. Similarly, gallic acid was most abundant in the n-butanol fraction (11.87 mg/g), moderately present in the ethyl acetate extract, and either absent or present in the other fractions, reinforcing the greater efficiency of polar solvents for extracting polar phenolic acids.

Cinnamic acid, on the other hand, exhibited low concentrations across most solvents but peaked in the ethyl acetate extract, indicating its preferential solubility in solvents with intermediate polarity. Chicoric acid was moderately concentrated in hydroethanolic and n-hexane extracts but was virtually absent elsewhere, reflecting a more complex solubility profile that may be influenced by structural features conducive to solubility in both polar and relatively non-polar environments.

Notably, quercetin was only detected in the n-hexane, dichloromethane, and ethyl acetate extracts, with ethyl acetate extracting the highest amount. The absence of quercetin in the hydroethanolic and n-butanol extracts suggests selectivity in solvent–phytochemical interactions, possibly due to the balance of polar and non-polar characteristics of quercetin.

The dichloromethane fraction uniquely contained significant amounts of 4-hydroxybenzoic acid and ferulic acid, compounds with moderate polarity that preferentially dissolve in solvents of intermediate polarity. Chlorogenic acid was exclusive to the n-butanol extract, consistent with its polar nature, whereas vanillic acid was found only in the ethyl acetate fraction, further emphasizing solvent selectivity

### 2.4. Antioxidant Activities

As shown in Table 4, the antioxidant activities of *E. angustifolia* leaf extracts were evaluated using ABTS, FRAP, and DPPH assays, revealing notable variation depending on the extraction solvent (Table 4).

The hydroethanolic extract showed moderate ABTS (13.98 ± 0.20 µg/mL) and DPPH (8.36 ± 0.09 µg/mL) activities and a FRAP value of 35.88 ± 2.00 µg/mL. The ethyl acetate extract exhibited the strongest ABTS activity (7.62 ± 0.17 µg/mL), while the dichloromethane extract demonstrated the highest FRAP (138.65 ± 0.45 µg/mL) and DPPH (58.13 ± 0.13 µg/mL) values. The n-butanol extract displayed moderate antioxidant activity across all assays, and the n-hexane extract showed comparatively weaker ABTS activity (41.15 ± 0.19 µg/mL) but strong FRAP and DPPH results, consistent with its composition. Among the standards, BHT, BHA, ascorbic acid, and α-tocopherol exhibited expected activities, with BHT and α-tocopherol showing strong DPPH scavenging, whereas BHA and ascorbic acid displayed generally lower activities. Statistical analysis using one-way ANOVA followed by Dunnett’s post hoc test indicated significant differences among all extracts and standards for each assay (*p* < 0.05), confirming that both solvent polarity and extract composition critically influence antioxidant performance. Overall, the hydroethanolic and ethyl acetate extracts demonstrated antioxidant activities comparable to or approaching those of the standard compounds, highlighting their potential as effective natural antioxidants.

### 2.5. In Vitro Antidiabetic Effect

According to the results of the antidiabetic effects presented in Table 5, the inhibitory activity of the different extracts increased in a concentration-dependent manner, with clear differences observed between samples. The n-hexane extract exhibited the highest inhibitory activity, with inhibition percentages increasing from 29.03 ± 1.27% at 6.25 µg/mL to 82.04 ± 1.71% at 400 µg/mL. It showed an IC_50_ value of 36.70 ± 0.92 µg/mL, indicating strong inhibitory potency, since lower IC_50_ values correspond to higher biological activity. This value was significantly different (*p* < 0.05) from dichloromethane (278.4 ± 3.13 µg/mL) and the standard acarbose (126.14 ± 10.70 µg/mL), as indicated by different superscript letters (a–c), confirming statistically significant differences among samples.

The dichloromethane extract demonstrated moderate inhibition, reaching 56.82 ± 0.27% at 400 µg/mL, but its high IC_50_ value reflects weaker potency. The standard drug acarbose showed intermediate activity compared to the tested extracts. In contrast, the hydroethanolic, ethyl acetate, and n-butanol extracts exhibited no detectable inhibition (ND) at the tested concentrations, with IC_50_ values > 400 µg/mL, indicating negligible activity under the experimental conditions.

All values are expressed as mean ± SD (n = 3). Statistical analysis was carried out using one-way ANOVA followed by Tukey’s post hoc test, and differences were considered significant at *p* < 0.05. Values with different superscript letters indicate statistically significant differences between groups.

### 2.6. In Silico Molecular Docking

**Figure 2:**
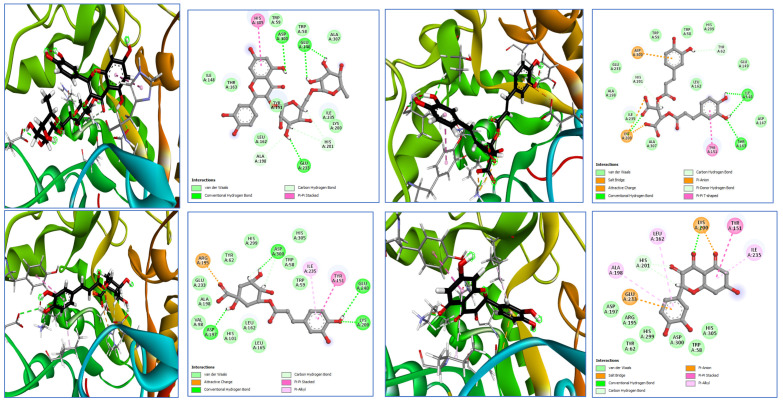
Two-dimensional and three-dimensional representations of the best docked compounds (rutin, chicoric acid, chlorogenic acid, and quercetin) within the active site of α-amylase (PDB ID: 3BAJ).

To investigate the potential antidiabetic activity of the compounds identified in *E. angustifolia*, molecular docking simulations were performed targeting α-amylase (PDB ID: 3BAJ). The docking results were compared with those of acarbose, a known α-amylase inhibitor, which served as the co-crystallized reference ligand. All compounds identified in the HPLC profiles (Figure 1) of the five extracts were subjected to docking analysis. However, only those exhibiting the highest binding affinities were retained for detailed interpretation, and the results are represented in Table 6 and Figure 2.

The co-crystallized ligand acarbose displayed the strongest binding affinity with a binding energy of −9.54 kcal/mol. It formed multiple hydrogen bonds with key active-site residues such as Tyr62, His299, Asp300, Arg195, Glu233, Lys200, Glu240, Gln63, Thr163, and Trp59. These interactions validate the reliability of the docking protocol.

Among the tested compounds, rutin exhibited the most promising interaction with α-amylase, with a binding energy of −8.66 kcal/mol. It formed hydrogen bonds with Asp300, Glu240, His201, Glu233, and Ala198, along with a hydrophobic interaction involving His305.

Chicoric acid demonstrated a binding energy of −7.82 kcal/mol and formed hydrogen bonds with residues such as Tyr62, His201, Lys200, Thr163, and Ile148. A hydrophobic interaction with Tyr151 was also observed. Chlorogenic acid exhibited a binding energy of −7.32 kcal/mol and formed strong hydrogen bonds particularly with Asp300, Lys200, Glu240, and Asp197, in addition to hydrophobic contacts with Ile235 and Tyr151. Quercetin, although less active, still formed a stable complex with α-amylase, with a binding energy of −6.71 kcal/mol. Its binding involved hydrogen interactions with Lys200 and His201 and hydrophobic contacts with Ile235, Tyr151, Leu162, and Ala198.

## 3. Discussion

According to Tourabi et al., 2025 [22], the observed differences in extraction yields can be primarily attributed to the varying polarities of the solvents used during maceration. Solvent polarity is a critical factor influencing the solubility of phytochemicals, such as phenolics, flavonoids, and other bioactive compounds present in *E. angustifolia* leaves. Polar solvents like hydroethanol are more effective in extracting polar compounds because of their ability to disrupt plant cell walls and solubilize hydrophilic constituents [23]. In contrast, non-polar solvents such as n-hexane tend to dissolve fewer polar constituents, resulting in lower extraction yields [24]. Additionally, solvent penetration and interaction with plant matrices depend on the dielectric constant and hydrogen bonding capacity [25]. The variation in extraction efficiency aligns with these physicochemical principles, explaining why solvents with higher polarity generally yield more extractable materials. These aspects emphasize the importance of solvent selection in optimizing extraction protocols to maximize bioactive compound recovery.

A comparative analysis of the various solvent fractions used to extract bioactive compounds from *E. angustifolia* revealed distinct differences primarily driven by solvent polarity and chemical affinity. Polar solvents such as hydroethanolic, ethyl acetate, and n-butanol generally exhibit higher efficiency in extracting phenolic compounds, flavonoids, and tannins compared than less polar solvents as such as n-hexane and dichloromethane [26]. This aligns with the principle of “like dissolves like,” as many phenolic and flavonoid molecules contain polar functional groups that facilitate the formation of hydrogen bonds with polar solvents, enhancing their solubility [26].

These observations correspond with findings from other studies, where polar organic solvents demonstrate superior extraction of polyphenolic compounds from *E. angustifolia* [27]. The efficiency differences among solvents can be explained by the chemical structures of the target compounds and their affinity for solvents of matching polarity [28]. Phenolic acids and flavonoids typically have multiple polar groups, favoring extraction by solvents capable of hydrogen bonding and polarity-based interactions, such as hydroethanolic and ethyl acetate mixtures. Conversely, non-polar solvents such as n-hexane are less effective because of their limited interactions with polar biomolecules [29,30].

Interestingly, the relatively high tannin content found in the dichloromethane fraction may be attributed to the structural complexity of tannins, which exist as both hydrolyzable and condensed forms with varying polarity. Some tannins possess moderately hydrophobic features, allowing partial solubility in solvents of intermediate polarity like dichloromethane [31,32,33]. This selective solubility illustrates that solvent polarity alone does not entirely determine extraction efficiency; the molecular size, degree of polymerization, and intramolecular hydrogen bonding within tannins also influence their affinity for specific solvents. Consequently, the dichloromethane fraction captures a unique profile of moderately polar tannins that are less accessible to highly polar or non-polar extraction media, highlighting the importance of tailored solvent selection to recover diverse bioactive constituents from *E. angustifolia* leaves.

Overall, the extraction profiles of *E. angustifolia* bioactive compounds were strongly influenced by solvent polarity and hydrogen bonding capabilities. Careful solvent selection based on the chemical nature of the phytochemicals is crucial for optimizing the recovery of phenolics, flavonoids, and tannins from *E. angustifolia* leaves.

Our findings add new information to the literature data on the *Elaeagnaceae* family. To the best of our knowledge, this is the first publication about the determination of the photochemical profile of the Algerian species *E angustifolia* via HPLC. The results proved the presence of bioactive compounds with interesting pharmacological activities. Therefore, this plant species could be identified as a potential source of compounds exhibiting antidiabetic and antioxidant properties.

The variations in the phenolic compound profiles observed across different *E. angustifolia* solvent extracts analyzed by HPLC are primarily due to the chemical structures of the compounds and their differential affinities for solvents with varying polarities. For instance, rutin, a flavonoid glycoside, contains multiple hydroxyl groups and sugar moieties, making it highly polar and capable of forming extensive hydrogen bonds [34]. This structural polarity explains the highest concentration of the compound in n-butanol solvent, which has a strong hydrogen bonding capacity, facilitating solubilization. Its absence in n-hexane or dichloromethane is due to their insufficient polarity to dissolve such hydrophilic compounds.

Similarly, gallic acid is a small phenolic acid with three hydroxyl groups that impart its pronounced polarity [35]. Their presence faciliates its effective extraction using polar solvents such as n-butanol and ethyl acetate, which can interact strongly with its polar phenolic groups. Its near absence in non-polar solvents aligns with its limited solubility in such media.

Cinnamic acid, which has a carboxyl group attached to an aromatic ring, exhibits moderate polarity. Therefore, it shows better extraction in solvents of intermediate polarity such as ethyl acetate, while being found only in low amounts in both highly polar and non-polar solvents [36]. Its amphiphilic nature allows some flexibility in solubility but generally favors solvents that balance polarity and hydrophobicity [37].

Furthermore, chicoric acid, a dicaffeoyltartaric acid derivative, possesses several polar hydroxyl and carboxyl groups as well as aromatic rings, giving it moderate-to-high polarity and hydrophobic characteristics [38]. Its extraction in both hydroethanolic (polar) and n-hexane (non-polar) solvents suggests structural features that allow partial solubility in both types of solvents, possibly because hydrophobic aromatic regions interact with less polar solvents and polar groups interact with polar solvents [39].

On the other hand, quercetin, a flavonoid aglycone without sugar attachments, is less polar than glycosides like rutin due to the absence of sugar moieties but still contains multiple hydroxyl groups [40]. It is preferentially extracted by medium-polarity solvents like ethyl acetate and dichloromethane, and to some extent n-hexane, reflecting its intermediate polarity and capability to interact with solvents that can balance polar and non-polar interactions [41]. The absence of highly polar solvents such as n-butanol may be due to the lower solubility of aglycones in very polar media.

Compounds such as 4-hydroxybenzoic acid and ferulic acid, detected mainly in the dichloromethane fraction, have moderate polarity and aromatic character. This favors extraction by solvents with intermediate polarity, which is consistent with their chemical structures that allow interaction via both hydrophobic and hydrogen bonding mechanisms [36].

Moreover, chlorogenic acid appeared only in the n-butanol extract, which can be explained by its highly polar ester linkage and multiple hydroxyl groups. These properties make it readily soluble in polar solvents [42].

Finally, vanillic acid, a methoxy-substituted hydroxybenzoic acid, was found exclusively in the ethyl acetate extract, indicating that its moderate polarity and hydrophobic character fit well with the polarity profile of this solvent.

Additionally, other factors can influence these extraction profiles, such as molecular size, possible formation of intramolecular hydrogen bonds that reduce polarity, and solvent viscosity, which affects diffusion rates. Moreover, the dielectric constant of the solvent and its ability to participate in specific interactions such as dipole–dipole and π-π stacking can preferentially stabilize certain compounds [43].

Thus, the selective extraction of phenolic compounds from *E. angustifolia* by different solvents is dictated by the intricate interplay of their chemical structures, polarity, hydrogen bonding capacity, and hydrophobic/hydrophilic balance. Understanding these relationships enables targeted extraction strategies to optimize yield of desired phytochemicals.

For the antioxidant activities of *E.angustifolia* leaf extracts, ABTS, FRAP, and DPPH assays revealed marked differences depending on the extraction solvent. The hydroethanolic and ethyl acetate extracts exhibited the strongest antioxidant potentials, which can be attributed to the presence of specific bioactive phenolic compounds, notably quercetin, ferulic acid, and 4-hydroxybenzoic acid [44]. These compounds are known for their potent radical scavenging and metal reducing properties, which likely contribute to the high performance in all three assays [45].

The n-butanol extract demonstrated moderate antioxidant capacity. This aligns with its relatively higher rutin and gallic acid content. These two compounds are widely recognized for their antioxidant potential, especially in aqueous or polar environments, which supports the observed activities [46]. The hydroethanolic extract also showed moderate effects, which can be correlated with the presence of rutin, gallic acid, and chicoric acid [47]. Although these phenolics were present at lower concentrations in the dichloromethane and n-hexane fractions, they still contributed synergistically to the antioxidant response. The differences observed among ABTS, DPPH, and FRAP results do indeed reflect the distinct reaction mechanisms and radical systems involved in each assay. The FRAP assay measures electron transfer capacity through Fe^3+^ reduction, the DPPH assay measures radical scavenging activity using a nitrogen-centered radical soluble in organic solvents, and the ABTS assay measures radical scavenging activity using a nitrogen-centered radical cation soluble in both aqueous and organic media [48,49,50]. These mechanistic differences, combined with differences in reaction pH, solvent, and sensitivity to steric effects, collectively explain the divergence among assay results for the same fraction [49,51]. The use of multiple complementary assays is therefore essential for a comprehensive characterization of antioxidant activity, as each assay captures a different dimension of the antioxidant capacity of phenolic-rich fractions [51].

Interestingly, the ethyl acetate extract, despite having the highest rutin concentration, displayed lower antioxidant activity. This might be explained by the limited diversity or synergistic interactions of phenolic compounds in this fraction, as well as possible degradation or reduced bioactivity in the solvent system. Additionally, the poor performance of certain standard antioxidants in some assays highlights the importance of assay specificity and the complex behavior of antioxidant molecules depending on their structure and polarity.

Overall, these findings suggest that the antioxidant potential of *E. angustifolia* extracts is closely linked to the total phenolic content and the specific composition and polarity of individual phenolic compounds. The variations observed between the solvent systems further underline the importance of solvent selection in optimizing the recovery of active antioxidants from plant materials.

For the antidiabetic effect, the observed α-amylase inhibitory activity of *E. angustifolia* leaf extract fractions, particularly the pronounced effect of the n-hexane and dichloromethane extracts, can be primarily attributed to the nature and chemical structures of the bioactive compounds selectively extracted by these solvents. The n-hexane fraction demonstrated the strongest inhibitory activity, which was significantly more potent than that of the dichloromethane fraction and far exceeded the activity of the hydrophilic fractions and the acarbose control.

The pronounced α-amylase inhibitory activity observed in the n-hexane fraction indicates that non-polar constituents may contribute to the enzyme inhibition. However, in the absence of direct analytical characterization of this specific fraction (e.g., GC-MS profiling), this interpretation remains tentative. Non-polar solvents such as n-hexane are known to preferentially extract lipophilic molecules, including fatty acids and terpenoid derivatives previously reported in *E. angustifolia* [52,53,54]. These types of compounds may interact with hydrophobic regions of α-amylase or influence its conformational stability [55]. Nevertheless, the precise constituents responsible for the observed inhibition have not yet been identified, and further phytochemical investigation and bioactivity-guided fractionation are required to confirm the active molecules

Dichloromethane, which is moderately polar, extracts a mixture of lipophilic and some moderately polar compounds. The lower inhibitory potency compared to n-hexane could be related to the increased presence of less active polar constituents or dilution of the active principles. Moreover, compounds such as certain phenolics with mid-range polarity could partially contribute to enzyme inhibition but less effectively than the non-polar molecules abundant in the n-hexane fraction.

On the other hand, the negligible inhibition observed with the hydroethanolic, n-butanol, and ethyl acetate fractions correlates with their polarity profiles, favoring the extraction of more polar phenolic acids, flavonoid glycosides, and tannins. While many polar phenolics are known for their antioxidant activity, their interaction with α-amylase is often weaker or requires different structural features (e.g., number and position of hydroxyl groups) to exhibit significant enzyme inhibition. It is also possible that these polar compounds are unable to bind effectively or sterically hinder the active site of α-amylase.

Additional factors influencing inhibitory activity include molecular size and shape, functional groups capable of forming non-covalent interactions (hydrogen bonding and van der Waals forces), and the overall three-dimensional conformation of the bioactive molecules. For example, certain small-molecule terpenoids or phenolic aglycones present in non-polar extracts may better fit into the α-amylase active site or allosteric sites, enhancing inhibition [56].

Moreover, synergistic effects among phytochemicals within the n-hexane and dichloromethane fractions could amplify enzyme inhibition beyond the sum of the activities of individual constituents. Conversely, the inhibitory activity might be diminished in polar fractions due to antagonistic interactions or the presence of compounds that stabilize enzyme activity.

Finaly, solvent residues and extract matrix effects can influence bioassay outcomes by altering enzyme stability or assay conditions, although these should be minimized through appropriate extraction and purification procedures.

Thus, the strong α-amylase inhibitory effects of n-hexane and dichloromethane extracts from *E. angustifolia* highlight the importance of solvent selection in accordance with phytochemical polarity to target specific bioactivities. These findings underscore the effectiveness of non-polar bioactive components in inhibiting α-amylase, potentially contributing to the antidiabetic potential of these extracts. Further phytochemical characterization would be valuable for identifying and isolating the active constituents responsible for this activity.

*E. angustifolia* extract contains significant amounts of fatty acids (like n-Hexadecanoic acid, 9-Octadecenoic acid, and 1,2,3-propanetriyl ester), phytol, alkaloids, squalene, and isoquinoline derivatives [53]. This may explain the strong α-amylase inhibitory potential observed for this plant.

Our molecular docking analysis showed that rutin, chicoric acid, chlorogenic acid, and quercetin, isolated from *E. angustifolia* L., bind within the active site of α-amylase (PDB ID: 3BAJ), although none surpassed the binding affinity of acarbose (−9.54 kcal/mol).

Rutin, one of the top-scoring compounds from *E. angustifolia* L., exhibited a binding energy of −8.66 kcal/mol in our docking simulations against α-amylase (PDB ID: 3BAJ), which was similar to the stability and affinity of acarbose (−9.54 kcal/mol). It formed hydrogen bonds with Asp300, Glu240, His201, Glu233, and Ala198, and a hydrophobic interaction with His305. These interactions suggest well-oriented and energetically favorable binding within the enzyme’s active site.

This in silico binding pattern echoes the findings of McMillan et al. [57], who identified rutin and acarbose as having among the best docking scores and highest stability when bound to pancreatic α-amylase and α-glucosidase. In their molecular dynamics simulations, rutin demonstrated conformational stability comparable to acarbose, and their in vitro assays showed that rutin’s Ki values for both α-amylase and α-glucosidase inhibition were not significantly different (*p* > 0.05) from those of acarbose, suggesting nearly equivalent inhibitory potency.

The relevance of rutin as a potent α-amylase and α-glucosidase inhibitor is further reinforced by the findings of Lekmine et al. (2023) [58], who demonstrated that *Hyoscyamus albus* L. extract, rich in rutin among other phenolics, exhibited strong in vitro inhibition of these enzymes, with a notably greater effect on α-amylase (IC_50_ = 146.63 µg/mL). Their molecular docking simulations also identified rutin as one of the top contributors to the observed inhibitory effects, corroborating our own results.

Furthermore, Bouslamti et al. [59] reported rutin as the most active phenolic compound against DPP-IV in *Solanum elaeagnifolium*, with a glide docking score of −8.10 kcal/mol, corroborating its strong enzyme binding profile. Although their focus was on DPP-IV, the notable involvement of residues equivalent to those we identified (e.g., Tyr151) reinforces a shared binding mechanism across carbohydrate-related enzymes.

Additionally, Ibrahim et al. [15] showed that in sweet basil extracts enriched in rutin via elicitation protocols, rutin exhibited significant α-amylase inhibitory activity. Molecular docking into porcine pancreatic α-amylase revealed hydrogen bonding and hydrophobic interactions consistent with strong binding, with reported binding energies around −5.74 kcal/mol, reflecting similar mechanistic features to those observed in our model

Chicoric acid exhibited a noteworthy binding affinity for α-amylase, as reflected by a docking score of −7.82 kcal/mol. Its interaction profile revealed the formation of multiple hydrogen bonds with key residues in the enzyme active site, including Tyr62, His201, Lys200, Thr163, and Ile148. In particular, Ile148 formed two strong hydrogen bonds at distances of 2.27 Å and 2.52 Å, suggesting a stable and specific interaction with the catalytic groove of the enzyme. Additionally, a hydrophobic interaction with Tyr151 further reinforced the binding stability of chicoric acid in the active pocket. Despite its promising in silico interaction with α-amylase, to the best of our knowledge, no previous study has reported or investigated the anti-amylase activity of chicoric acid. This lack of data in the current scientific literature positions our findings as a novel contribution to the field. Given the strong binding of chicoric acid to several residues implicated in enzymatic catalysis and substrate recognition, chicoric acid has emerged as a potential natural α-amylase inhibitor. These results warrant further experimental validation, both in vitro and in vivo, to confirm the inhibitory potential of chicoric acid and explore its applicability in the management of hyperglycemia and related metabolic disorders.

Chlorogenic acid, one of the major compounds identified in *E. angustifolia* demonstrated a binding energy of −7.32 kcal/mol in our docking experiments against α-amylase (PDB ID: 3BAJ). The interaction profile revealed that chlorogenic acid formed multiple hydrogen bonds and hydrophobic contacts with key catalytic residues within the active site of α-amylase, notably Asp197, Glu233, and Asp300. These interactions are consistent with the mechanisms underlying enzymatic inhibition, supporting the plausibility of chlorogenic acid as a competitive inhibitor.

This computational observation aligns with previous experimental findings that documented the α-amylase inhibitory activity of chlorogenic acid. In particular, Narita and Inouy [60] demonstrated that chlorogenic acid from green coffee beans significantly inhibited porcine pancreatic α-amylase in a dose-dependent manner, with IC_50_ values of 9.10 μg/mL and 9.24 μg/mL, respectively. These results suggest that chlorogenic acid can modulate postprandial glucose levels by limiting starch breakdown, a property that is highly desirable in the context of type 2 diabetes management. Furthermore, the ability of chlorogenic acid to inhibit α-amylase has been corroborated by other studies that emphasize its role in reducing carbohydrate digestion and glucose absorption. For instance, Santana-Gálvez et al. (2017) [61] highlighted the antidiabetic potential of chlorogenic acid-rich extracts, attributing part of their activity to α-amylase inhibition.

More recently, Zheng et al. (2020) [62] investigated porcine pancreatic α-amylase and identified chlorogenic acid (CHA) as a mixed-type inhibitor of the enzyme, with an IC_50_ of 0.498 ± 0.013 mg/mL. Their spectroscopic and circular dichroism analyses confirmed that CHA altered the enzyme’s secondary structure via hydrogen bond interactions, and docking studies revealed binding energies around −7.8 to −7.2 kcal/mol at both active and secondary sites, values consistent with our calculated binding affinity. Similarly, Cardullo et al. (2021) [63] showed that CA can inhibit both α-amylase and α-glucosidase, thereby slowing intestinal glucose absorption. To enhance its activity, the authors synthesized eleven CA-derived amides, among which two compounds (amides 8 and 11) exhibited significantly stronger inhibitory effects than native CA, even surpassing acarbose in α-glucosidase inhibition. Kinetic studies revealed that these compounds act through mixed or competitive inhibition mechanisms depending on the enzyme. Molecular docking further supported the enhanced binding affinity, as these derivatives formed additional interactions with the enzyme active sites. Notably, benzothiazole-based amide 11 showed a synergistic inhibitory effect when combined with acarbose, suggesting its promising therapeutic potential.

Quercetin displayed a binding energy of −6.71 kcal/mol, forming hydrogen bonds with Lys200 and His201 and hydrophobic contacts with Ile235, Tyr151, Leu162, and Ala198. These interactions align well with the evidence presented by Günal-Köroğlu et al. (2024) [64], who reported the hypoglycemic activity of quercetin with IC_50_ values comparable to those of acarbose, highlighting hydrogen bond formation with key active-site residues via docking, MD simulations, and QSAR modeling to explain the mechanism of carbohydrate digestion inhibition and reduction in glucose absorption. Similarly, Raut et al. (2023) [65] identified quercetin as the most potent inhibitor in a polyphenolic library using molecular docking, MM/GBSA, and 100 ns MD simulations, confirming strong hydrogen bonding with the catalytic triad of human pancreatic α-amylase and a favorable free energy of −27.03 kcal/mol. Their in vitro data further validated the effectiveness of quercetin, with an IC_50_ of 57.37 ± 0.9 µg/mL. In a spectroscopic study by Huang et al. (2024) [66], quercetin was shown to be a mixed-type inhibitor of α-amylase. The authors observed static quenching of tryptophan and tyrosine residues, reflecting conformational alterations, and confirmed hydrogen bonds with Asp197 and Gln63, along with hydrophobic interactions involving Ala198, Leu165, and Trp59. This finding supports the multifaceted binding observed between His201 and Lys200. Furthermore, Shen et al. (2023) [67] reported that quercetin acts via non-competitive inhibition, with an IC_50_ of 0.325 mg/mL. Their combined spectroscopic (UV-vis, FTIR, and CD), kinetic, docking and MD analyses showed hydrogen bonding with Asp197, Glu233, and Asp300, as well as stable enzyme–quercetin complexes, consistent with the critical active-site contacts. Altuner et al. (2022) [68] further corroborated the inhibitory potential of quercetin through in silico docking, LigPlot+ analysis, and ADMET profiling. They visualized the strong interactions of quercetin within the α-amylase binding site and highlighted its favorable pharmacokinetic and drug-likeness properties when compared to acarbose [68].

In silico molecular docking was performed on selected phenolic compounds identified in *E. angustifolia* to explore their potential interactions with α-amylase. Chicoric acid, rutin, and other phenolics exhibited favorable binding poses and interactions within the enzyme active site, suggesting possible mechanistic contributions to enzyme inhibition. It should be noted, however, that docking scores are predictive and do not provide experimental validation of binding affinity. Interestingly, the n-hexane extract showed the strongest α-amylase inhibitory activity experimentally, despite lacking the phenolic compounds included in the docking study. This discrepancy is likely due to the presence of lipophilic constituents (e.g., terpenoids, fatty acids) in the non-polar n-hexane fraction, which were not included in the docking panel. Furthermore, inhibition observed in complex extracts may result from synergistic or additive effects among multiple compounds. Therefore, while docking provides mechanistic insights for selected phenolics, it does not fully account for the bioactivity of the complete extracts, particularly non-polar fractions such as n-hexane.

## 4. Materials and Methods

### 4.1. Collection, Handling, and Extraction of Botanical Sample

*E. angustifolia* leaves were collected from the Baghaï region (Khenchela, Algeria) and taxonomically authenticated by Prof. Zereib Azzedine (Department of Agronomy, Abbas Laghrour University, Khenchela). A voucher specimen (EA-01) was deposited at the Herbarium of the Faculty of Natural Sciences, Abbas Laghrour University, Khenchela, Algeria, for future reference. The collection and identification of plant material were conducted in accordance with institutional guidelines, and the voucher specimen is available for consultation upon reasonable request.

Fresh leaves were air-dried at room temperature (25 ± 2 °C) under shade and protected from direct sunlight to prevent phytochemical degradation. The dried material was mechanically ground into a fine powder and stored in airtight containers under dry, light-protected conditions until extraction. For extraction, 100 g of powdered plant material was macerated in 80% ethanol (ethanol:water, 80:20 *v*/*v*) at a 1:10 (*w*/*v*) ratio. The extraction was performed under continuous magnetic stirring in darkness at room temperature for 96 h to minimize oxidative degradation. The mixture was filtered through Whatman No. 1 filter paper, and the solvent was removed under reduced pressure using a rotary evaporator at 40 °C.

The resulting crude hydroethanolic extract was suspended in distilled water and sequentially partitioned with solvents of increasing polarity: n-hexane, dichloromethane, ethyl acetate, and n-butanol. Each organic fraction was concentrated under reduced pressure at 40 °C and stored at 4 °C until further phytochemical and biological analyses.

### 4.2. Determination of Total Phenolics, Flavonoids, and Condensed Tannins

The total phenolic, flavonoid, and condensed tannin contents of *E. angustifolia* extracts were quantified using microplate-based colorimetric methods. Total phenolics were assessed following the Folin–Ciocalteu procedure described by Singleton and Rossi (1965) [69], with absorbance recorded at 765 nm and results expressed as µg gallic acid equivalents per mg extract (µg GAE/mg). Total flavonoids were determined using a modified aluminum chloride assay according to Topçu et al. (2007) [52], measuring absorbance at 415 nm and expressing results as µg quercetin equivalents per mg extract (µg QE/mg). Condensed tannins were evaluated using the vanillin–HCl method adapted from Serif et al. (2023) [70], with absorbance read at 500 nm and values reported as µg catechin equivalents per mg extract (µg CE/mg).

### 4.3. High-Performance Liquid Chromatography (HPLC)

Phenolic acids in *E. angustifolia* extracts were analyzed by high-performance liquid chromatography (HPLC) with a diode array detector (Shimadzu, Kyoto, Japan; LC-20AT pump, SPD-M20A DAD). Separation was achieved on a reverse-phase Dionex Acclaim Polar Advantage C16 column (150 × 4.6 mm, 3 µm) (Thermo Fisher Scientific, Kyoto, Japan) using a gradient of methanol (A) and 1% formic acid in water (B): 0 min (100% A), 10 min (60% A), 17.5 min (100% B) for washing and re-equilibration. The flow rate was 1 mL/min, the column temperature was 30 °C, and detection occurred at 280 nm. Individual acids—including gallic, protocatechuic, p-hydroxybenzoic, vanillic, syringic, caftaric, caffeic, p-coumaric, ferulic, chicoric, rosmarinic, and cinnamic acids—were identified by matching retention times and UV spectra to authentic standards.

Quantification was performed using external calibration curves prepared with pure standard compounds at seven concentrations. The calibration curves showed excellent linearity with correlation coefficients (R^2^) ≥ 0.995 for all phenolics. The limits of detection (LODs) and quantification (LOQs) were determined based on signal-to-noise ratios of 3:1 and 10:1, respectively, and ranged from 0.05 to 0.2 mg/kg for LODs and 0.15–0.6 mg/kg for LOQs, depending on the compound. Recovery studies were conducted by spiking known amounts of standards into the extracts, and recoveries ranged from 94 to 103%, demonstrating the accuracy and reliability of the method. The amounts of phenolic acids in the samples were then calculated from the calibration curves and are expressed in mg/kg dry weight.

### 4.4. Antioxidant Activity

The antioxidant potential of *E. angustifolia* extracts was evaluated using DPPH, ABTS, and reducing-power assays. For DPPH and ABTS scavenging, 40 µL of each extract at varying concentrations was incubated with the respective radicals (160 µL) in the dark, and absorbance was measured at 517 nm (DPPH) or 734 nm (ABTS) to calculate IC50 values, using BHA, BHT, Trolox, and ascorbic acid as standards [71,72]. Reducing power was determined by mixing 10 µL of extract with phosphate buffer and potassium ferricyanide, followed by incubation at 50 °C and reaction with trichloroacetic acid, water, and ferric chloride; absorbance at 700 nm was recorded, and A0.5 values were calculated [73]. All assays were performed in triplicate, and the results reflect the concentration required for 50% radical inhibition or an absorbance of 0.5, indicating antioxidant strength.

### 4.5. Alpha-Amylase Antidiabetic Activity Bioassay

The α-amylase inhibitory activity of *E. angustifolia* extracts was evaluated following the method described by Zengin et al. (2014) [74].

Porcine pancreatic α-amylase (EC 3.2.1.1, Sigma-Aldrich, Saint Louis, MO, USA) was used as the enzyme source and prepared at a concentration of 10 U/mL in phosphate buffer (20 mM, pH 6.9, containing 6 mM NaCl).

Briefly, 25 µL of plant extract (1 mg/mL) was mixed with 50 µL of α-amylase solution and pre-incubated at 37 °C for 10 min to allow enzyme–inhibitor interaction. Subsequently, 50 µL of 0.1% (*w*/*v*) soluble starch solution (prepared in the same phosphate buffer) was added as substrate, and the mixture was incubated at 37 °C for 10 min.

The reaction was terminated by adding 25 µL of 1 M HCl. Then, 100 µL of iodine–potassium iodide reagent (I_2_/KI solution) was added to detect residual starch. After 10 min of incubation at 37 °C, absorbance was measured at 630 nm using an EnSpire Multimode Plate Reader (PerkinElmer, Springfield, IL, USA).

Acarbose was used as a positive control under identical conditions.

The following controls were included:

Control (Ac): enzyme + substrate without extract

Sample (As): enzyme + substrate + extract

Blank (Ab): extract + substrate without enzyme

Enzyme blank (Ae): enzyme without substrate

The percentage of α-amylase inhibition was calculated using the equation

$$\alpha \text{-}\mathrm{Amylase}\;\mathrm{inhibition}\;(\%)=1-\frac{\left(A_{c}-A_{e})-(A_{s}-A_{b}\right)}{A_{c}-A_{e}}$$

### 4.6. Statistical Analysis

The statistical analysis of the data was conducted utilizing GraphPad Prism DataEditor 6.0. Initially, one-way ANOVA was employed to analyze the data, followed by Dunnett’s test to identify significant differences between the tests. The results were expressed as the mean ± standard error of the mean. A *p*-value of less than 0.05 was considered statistically significant, indicating meaningful differences between the experimental groups. This comprehensive analysis allowed for the accurate interpretation of the data and the identification of significant outcomes within the study.

### 4.7. In Silico Molecular Docking

Major phytochemicals identified in *E. angustifolia* extracts by HPLC were selected for molecular docking studies. Their 3D structures were retrieved from PubChem and converted to Mol2 format using Open Babel [75]. Ligand preparation was performed in AutoDock Tools [76]. by adding Gasteiger charges, merging non-polar hydrogens, and defining rotatable bonds before saving the structures as PDBQT files.

The crystal structure of human pancreatic α-amylase (PDB ID: 3BAJ) was obtained from the Protein Data Bank [76]. Specifically, the structure corresponds to human pancreatic α-amylase in complex with an inhibitor [77]. Water molecules and heteroatoms were removed, polar hydrogens were added, and Kollman charges were assigned using AutoDock Tools.

Docking simulations were carried out using AutoDock 4.2.6 [76]. employing the Lamarckian Genetic Algorithm. A grid box was centered on the enzyme active site to ensure adequate sampling of ligand conformations within the catalytic pocket. Each ligand was subjected to 50 independent docking runs, and the best binding pose was selected based on the lowest binding energy and clustering analysis.

Binding modes and molecular interactions, including hydrogen bonds and hydrophobic contacts, were visualized and analyzed using Discovery Studio Visualizer (BIOVIA, 2019).

## 5. Conclusions

Collectively, these in vitro results establish a direct relationship between solvent polarity, phenolic compound profiles, and the respective antioxidant and antidiabetic activities of the extracts. This underscores the critical importance of optimizing extraction solvents to maximize the yield of therapeutically valuable phytochemicals. The study highlights, for the first time, the potent in vitro α-amylase inhibitory activity of non-polar leaf extracts from *E. angustifolia*, thereby supporting its potential as a promising source for the discovery of novel antidiabetic agents. However, these findings are preliminary and necessitate future bioassay-guided fractionation to isolate the pure active compound(s), followed by detailed in vivo pharmacological and toxicological studies to truly validate its therapeutic potential in managing diabetes and its complications.

## Figures and Tables

**Table 1:** Extraction yields of *E. angustifolia* leaf powder using different solvents.

Solvent	Extraction Yield (%)
Hydroethanolic	18.00
N-Butanol	4.32
Ethyl acetate	3.84
Dichloromethane	0.32
N-Hexane	0.05

**Table 2:** Total phenol, flavonoid, and tannin contents of *E. angustifolia* leaf powder extracts using different solvents.

Solvent	Total Phenol Content (mg GAE/g)	Flavonoid Content (mg QE/g)	Tannin Content (mg CE/g)
Hydroethanolic	244.09 ± 2.07 ^c^	53.90 ± 0.41 ^c^	25.58 ± 0.36 ^a^
N-Hexane	92.23 ± 0.65 ^a^	37.77 ± 0.30 ^b^	22.26 ± 0.92 ^a^
Dichloromethane (DCM)	136.44 ± 0.64 ^b^	40.28 ± 0.42 ^b^	66.24 ± 0.79 ^c^
Ethyl Acetate	290.97 ± 1.21 ^c^	102.11 ± 0.79 ^d^	30.71 ± 0.25 ^b^
N-Butanol	309.05 ± 0.64 ^d^	29.33 ± 0.58 ^a^	25.58 ± 0.67 ^a^

Different superscript letters (a–d) within the same column indicate statistically significant differences (*p* < 0.05). Values are mean ± standard deviation (n = 3). GAE = gallic acid equivalents; QE = quercetin equivalents; CE = catechin equivalents.

**Table 3:** Identification and quantification of major phenolic compounds in *E. Angustifolia* leaf extracts obtained with different solvents using HPLC analysis.

	Hydroethanolic			N-Hexane			Dichloromethane			N-Butanol			Ethyl Acetate		
Compound	RT	Area	Conc.	RT	Area	Conc.	RT	Area	Conc.	RT	Area	Conc.	RT	Area	Conc.
Rutin	22.599	137,091	6.748 ± 0.21	ND	ND	ND	ND	ND	ND	22.616	309,807	15.250 ± 0.45	22.898	1,539,237	75.768 ± 1.32
Gallic Acid	4.044	34,380	1.048 ± 0.05	ND	ND	ND	ND	ND	ND	4.828	46,950	1.431 ± 0.07	4.003	389,470	11.873 ± 0.54
Cinnamic Acid	31.069	3617	0.041 ± 0.003	ND	ND	ND	30.296	2526	0.028 ± 0.002	5.620	1291	0.014 ± 0.001	30.012	52,781	0.592 ± 0.021
Chicoric Acid	27.077	3686	0.333 ± 0.012	27.030	10,046	0.906 ± 0.034	ND	ND	ND	27.377	1815	0.164 ± 0.006	ND	ND	ND
Quercetin	ND	ND	ND	32.561	9180	0.296 ± 0.011	32.113	3246	0.105 ± 0.004	ND	ND	ND	32.373	83,099	2.676 ± 0.09
4-Hydroxybenzoic Acid	ND	ND	ND	ND	ND	ND	10.637	98,936	1.508 ± 0.06	ND	ND	ND	ND	ND	ND
Ferulic Acid	ND	ND	ND	ND	ND	ND	20.057	230,241	3.834 ± 0.14	ND	ND	ND	ND	ND	ND
Chlorogenic Acid	ND	ND	ND	ND	ND	ND	ND	ND	ND	11.921	17,459	0.524 ± 0.018	ND	ND	ND
Vanillic Acid	ND	ND	ND	ND	ND	ND	ND	ND	ND	ND	ND	ND	12.598	32,291	0.684 ± 0.023

Concentration values of major phenolic compounds in *E. angustifolia* leaf extracts obtained with different solvents are presented as mean ± SD (n = 3). The symbol “ND” indicates that the compound was not detected in the corresponding extract. Standard deviations reflect variability among triplicate measurements.

**Table 4:** Antioxidant activities of *E. angustifolia* leaf extracts evaluated by ABTS, FRAP, and DPPH assays.

Extract	ABTS (µg/mL)	FRAP (µg/mL)	DPPH (µg/mL)
Hydroethanolic	13.98 ± 0.20 ^c^	35.88 ± 2.00 ^b^	8.36 ± 0.09 ^c^
N-hexane	41.15 ± 0.19 ^f^	103.04 ± 1.83 ^e^	35.55 ± 0.11 ^h^
Dichloromethane	36.63 ± 3.31 ^e^	138.65 ± 0.45 ^f^	58.13 ± 0.13 ^i^
Ethyl acetate	7.62 ± 0.17 ^b^	41.31 ± 0.17 ^c^	17.31 ± 0.29 ^f^
N-butanolic	30.66 ± 0.81 ^d^	81.32 ± 0.96 ^d^	29.71 ± 0.12 ^g^
BHT	1.29 ± 0.30 ^a^	/	12.99 ± 0.15 ^e^
BHA	1.81 ± 0.10 ^a^	8.41 ± 0.67 ^a^	6.14 ± 0.20 ^b^
Ascorbic	/	9.01 ± 1.46 ^a^	3 ± 1.12 ^a^
α-Tocopherol	/	34.93 ± 2.38 ^b^	13.02 ± 5.17 ^d^

Different superscript letters (a–i) within the same column indicate statistically significant differences (*p* < 0.05).

**Table 5:** α-Amylase inhibitory activity of different *E. angustifolia* leaf extract fractions.

Samples	6.25 µg/mL	12.5 µg/mL	25 µg/mL	50 µg/mL	100 µg/mL	200 µg/mL	400 µg/mL	IC_50_ (µg/mL)
Hydroethanolic	ND	ND	ND	ND	ND	ND	ND	>400
n-Hexane	29.03 ± 1.27	34.44 ± 2.24	43.78 ± 0.16	58.06 ± 0.27	68.78 ± 3.53	79.74 ± 0.90	82.04 ± 1.71	36.70 ± 0.92 ^a^
Dichloromethane	14.50 ± 1.81	18.74 ± 1.37	18.14 ± 2.88	24.75 ± 2.08	35.89 ± 0.55	40.66 ± 2.45	56.82 ± 0.27	278.4 ± 3.13 ^c^
Ethyl acetate	ND	ND	ND	ND	ND	ND	ND	>400
n-Butanol	ND	ND	ND	ND	ND	ND	ND	>400
Acarbose	29.76 ± 0.17	33.36 ± 0.30	39.46 ± 0.11	43.68 ± 0.96	48.76 ± 0.17	57.36 ± 0.30	69.46 ± 0.11	126.14 ± 10.70 ^b^

Different superscript letters (a–c) within the same column indicate statistically significant differences (*p* < 0.05). Values are expressed as mean ± SD (n = 3). ND = not detected.

**Table 6:** Molecular docking results of the most active compounds identified from HPLC profiles of *E. angustifolia*. extracts against α-amylase (PDB ID: 3BAJ).

Target	Compound	Binding Energy (kcal/mol)	Ki (µM)	Hydrogen Interactions (Å)	Hydrophobic Interactions
α-Amylase (3BAJ)	Acarbose (co-crystallized)	−9.54	0.10	Tyr62, His299, Asp300, Arg195, Glu233, Lys200, etc.	–
	Rutin	−8.66	0.45	Asp300, Glu240, His201, Glu233, Ala198	His305
	Chicoric acid	−7.82	1.90	Tyr62, His201, Lys200, Thr163, Ile148	Tyr151
	Chlorogenic acid	−7.32	4.40	Lys200, Glu240, Asp300, Asp197	Ile235, Tyr151
	Quercetin	−6.71	12.1	Lys200, His201	Ile235, Tyr151, Leu162, Ala198

## Data Availability

The original contributions of this study are available in the article. For additional information, please contact the corresponding authors.
